# Development of a Prospective Data Registry System for Non-muscle-Invasive Bladder Cancer Patients Incorporated in the Electronic Patient File System

**DOI:** 10.3389/fonc.2019.01402

**Published:** 2019-12-11

**Authors:** Murat Akand, Tim Muilwijk, Jan Cornelissen, Siska Van Bruwaene, Kathy Vander Eeckt, Frederic Baekelandt, Pieter Mattelaer, Raf Van Reusel, Ben Van Cleynenbreugel, Steven Joniau, Frank Van Der Aa

**Affiliations:** ^1^Department of Urology, University Hospitals Leuven, Leuven, Belgium; ^2^Department of Urology, School of Medicine, Selçuk University, Konya, Turkey; ^3^Department of Urology, AZ Groeninge, Kortrijk, Belgium; ^4^Department of Urology, AZ Sint Blasius, Dendermonde, Belgium; ^5^Department of Urology, AZ Sint Lucas, Bruges, Belgium; ^6^Department of Urology, AZ Damiaan Oostende, Oostende, Belgium; ^7^Department of Urology, AZ Turnhout, Turnhout, Belgium

**Keywords:** bladder cancer, transurethral resection of bladder tumor, database, registry, patient flow

## Abstract

**Purpose:** To develop a prospective non-muscle-invasive bladder cancer (NMIBC) data registry by generating NMIBC-specific electronic case report forms (eCRFs) in our institution's electronic patient file system, and to report on the development and implementation of a prospective multicentric registry.

**Methods:** Templates for data collection, including clinical outcome parameters and quality indicators, were developed in InfoPath™ as an eCRF and were incorporated in our hospital's electronic patient file system. Quality parameters for managing NMIBC patients that were identified by comprehensive literature review were included in the eCRFs. Three separate eCRFs were developed for the management of NMIBC patients: surgery report, bladder instillation form, and multidisciplinary team form.

**Results:** In August 2013, we started a Flemish prospective clinical and pathological data registry for all patients undergoing transurethral resection of bladder tumor (TURBT) for NMIBC in four participating hospitals, three of which continued using this to date. Three more hospitals started enrolling in 2017, 2018, and 2019, respectively. Written reports of the registered clinical actions are automatically generated within the electronic medical file. When urologists complete these eCRFs, an automated ready-to-send letter to the general practitioner is generated. Up till May 2019, 2,756 TURBTs in 2,419 patients are included in the dataset. Currently, we are recruiting over 600 TURBTs every year.

**Conclusions:** Easy-to-use eCRFs were developed and included in the electronic patient file system. This registration tool was implemented in 7 hospitals, 6 of which are still using it today. The register harvests important clinical data, while performing routine clinical practice. The data will be used to analyze real-life data of NMIBC patients, to challenge the existing guidelines, to create novel risk stratification tools, and to develop, monitor and validate quality parameters for NMIBC management.

## Introduction

Bladder cancer (BC) is a major health problem, as it is the ninth most commonly diagnosed form of cancer, and accounts for 3% of all cancer-related deaths in Europe (1). The incidence and prevalence increase with age. At first diagnosis, the large majority of detected lesions (±75%) are classified as non–muscle-invasive bladder cancer (NMIBC). These superficial lesions are defined as Ta, T1, or carcinoma *in situ* (CIS). The primary treatment for NMIBC is the removal of all cancerous tissue from the bladder, called transurethral resection of bladder tumor (TURBT), which is used both as a diagnostic and therapeutic tool. Up to 70% of the NMIBC cases will recur, and 15% of all cases will progress in stage and grade (2). Therefore, accurate and early diagnosis of NMIBC is essential to offer the patients the most appropriate treatment and the highest cure rate. For the same reason, NMIBC patients are scheduled to undergo frequent monitoring, currently based on cystoscopy and cytology, which makes BC one of the costliest of all cancers to manage (3, 4).

TURBT is often considered as a straightforward and easy-to-do procedure, and is therefore often treated like a Cinderella (e.g., leaving the procedure to resident). Data suggest that there is wide variability in the quality of TURBTs performed in different centers (5). Several recommendations have been made for modifying the TURBT technique with the ultimate aim to increase its quality (6–8). Although three TURBT checklists have been proposed to improve the quality of the operation, only the 10-item one developed by Anderson et al. has been evaluated in clinical practice (9–12).

Current guidelines are based on relatively small prospective patient cohorts with medium-term follow-up. The European Organization for Research and Treatment of Cancer (EORTC) risk calculator, which predicts the short- and long-term risks of disease recurrence and progression, is the result of a *post-hoc* statistical analysis of 2,596 patients, treated between 1979 and 1989, from seven separate prospective trials with 291 to 517 included patients (13). They were categorized by the old (pre-2004) WHO grading system. Because only a minority (*n* = 171) patients in the EORTC cohort were treated with Bacillus Calmette–Guérin (BCG) and none of them received maintenance treatment (which is now considered mandatory for at least 12 months to lead to an effect), the Spanish CUETO consortium (Club Urologico Español de Tratamiento Oncologico) developed another risk stratification model that predicts the risk of recurrence and progression based on a total of 1,062 patients treated with BCG between February 1990 and May 1999 in 4 prospective trials (14). Both risk calculators tend to overestimate the risk of disease recurrence and progression in high-risk patients and have poor discrimination for prognostic outcomes in external validation (15, 16).

Based on the known risk factors, NMIBC patients are stratified into three risk categories: low-, intermediate- and high-risk. Treatment recommendations are guided by this stratification (17). Management of intermediate- and high-risk NMIBC consists of TURBT and bladder instillations with BCG plus intensive follow-up and maintenance BCG. Despite this intensive treatment and follow-up schedule, these patients have a high risk for disease recurrence and a moderate to high risk for progression to muscle-invasive bladder cancer (MIBC) of up to 35–55% at 5-year follow-up (17).

The care of NMIBC patients is complex. Even in modern medicine, concerns have been raised regarding the variation in management of patients with BC (18). Population based data have shown the real-life survival is lower than expected from clinical trials (19, 20). A clear patient flow chart with predefined outcome parameters and quality indicators is expected to improve overall patient care. The current major challenge and unmet need is prospective real-life collection of NMIBC patient data. There is need for robust reporting rules and robust internally and externally validated prediction models based on up-to-date datasets. As timely updating of the currently used and above-mentioned datasets is impossible (as they are *post-hoc* analyses of terminated trials), a prospective dataset needs to be developed.

Keeping these unmet needs and deficiencies of the former risk stratifications in mind, we developed a prospective NMIBC data registry by generating NMIBC-specific electronic case report forms (eCRFs). With this registry, we aimed to benchmark the current standard of care with existing guidelines, and also collect high-quality data to develop a novel prediction model. In this manuscript, we report on the development of these eCRFs and their implementation in a prospective multicentric registry.

## Materials and Methods

Electronic forms for data collection, including relevant clinical outcome parameters and quality indicators, were developed in InfoPath™ (Microsoft Corporation, Redmond USA). InfoPath forms serve as interface in front of the electronic patient file system. These forms are used as an eCRF. To comply with local privacy laws, all data is stored in the hospital's electronic patient file system itself (called Klinisch Werkstation (KWS), which runs in different Flemish hospitals), which is protected by firewalls. The eCRFs have been developed based on the recommendations of the European Association of Urology (EAU) guidelines and the Canadian Urological Association (CUA) white paper by consensus of two academic and one non-academic urologists (17, 18).

Three separate eCRFs were developed for the management of NMIBC patients: a surgery report form, bladder instillation form, and multidisciplinary team (MDT) form. The data collected in these forms are listed in Tables 1–3, respectively. Besides scientific outcome parameters and quality indicators, specific attention was paid to patient comorbidities by systematically including Charlson Comorbidity Index and smoking status. These eCRFs serve for daily clinical practice and for prospective data registration at the same time. With automatically generated ready-to-send letters, they provide standardized data collection while not increasing the workload of the urologist. Data is extracted trough algorithms with pseudonymization for centers and anonymized for intercenter sharing. This registry has been registered in ClinicalTrials.gov (NCT03973671).

**Table 1:** The data collected in the operation form.

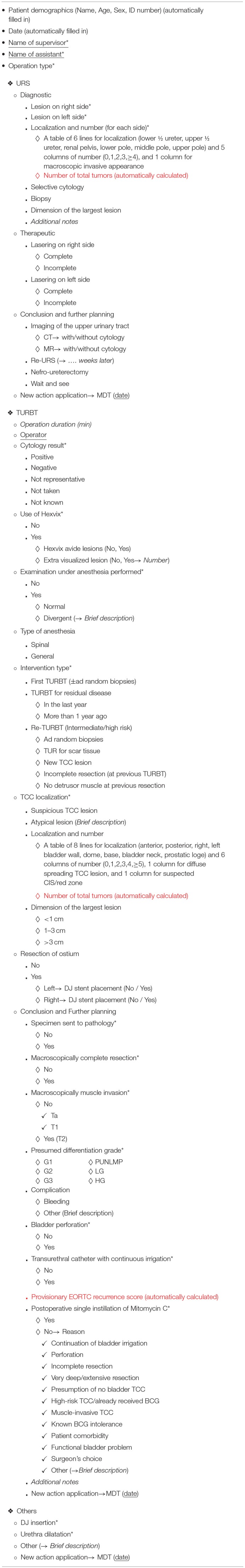

Underlined parameters are chosen from the drop-down menus. Parameters in italics need to be written manually. Automatically filled in and automatically calculated parameters are mentioned in parenthesis, and are colored in black and red, respectively. Answers for all other parameters are clicked from the options listed below the parameters. Items marked with an asterisk (*) are mandatory fields.

**Table 2:** The data collected in the instillation form.

Patient demographics (Name, Age, Sex, ID number) (automatically filled in) Date (automatically filled in) Name of supervisor Name of assistant Name of nurse Instillation type^*^ ∘ Postoperative single instillation of MMC ∘ Induction schema for chemotherapy ∘ Maintenance treatment for chemotherapy ∘ Induction schema for BCG ∘ Maintenance treatment for BCG ∘ Interstitial cystitis/Bladder pain syndrome/Radiocystitis Catheter type and dimension Session number^*^ Instillation product and dosage^*^ ∘ Chemotherapy ▪ Mitomycin C (40 mg, 1/2, 1/3) ▪ Epirubicine (50 mg, 80 mg, 1/2) ▪ Doxorubicine (50 mg, 1/2, 1/3) ▪ Gemcitabine (2 g, 1/2, 1/3) ∘ BCG ▪ OncoTice (12.5 mg, 1/2, 1/3) ▪ BCG Medac (50 mL, 1/2, 1/3) ▪ Immunocyst (81 mg, 1/2, 1/3) Instillation not administered ∘ Reason^*^ ▪ Suspicion of UTI ▪ Hematuria ▪ Bladder perforation ▪ According to doctor's advice ▪ MMC not delivered ▪ Patient did not come ▪ Patient's intolerance ∘ Date of the new instillation Residual urine beforehand ∘ No ∘ Yes→ *Volume (ml)* Patient's complaints^*^ ∘ No ∘ Yes ▪ Macroscopic hematuria ▪ UTI ▪ LUTS (without a sign of infection) ▪ Fever ▪ Dyspnea ▪ Arthralgia ▪ Other (*Brief description*) Dipstick test performed ∘ No ∘ Yes ▪ Leucocyte esterase ♢ Positive ♢ Negative ▪ Nitrite ♢ Positive ♢ Negative Post-instillation ∘ How did the instillation go^*^ ▪ Smooth ▪ Troublesome but atraumatic catheterization ▪ Traumatic catheterization ∘ Experience of pain (from 1 to 10) ∘ Urine culture taken/performed ▪ Yes ▪ No ∘ Presence of residue afterwards ▪ Yes ▪ No ∘ Planned next instillation ▪ Yes→ Date ▪ No→ Reason ♢ End of schema ♢ Intolerance ♢ Traumatic catheterization ♢ Due to contraindication

Underlined parameters are chosen from the drop-down menus. Parameters in italics need to be written manually. Automatically filled in and automatically calculated parameters are mentioned in parenthesis, and are colored in black and red, respectively. Answers for all other parameters are clicked from the options listed below the parameters. Items marked with an asterisk

(*) *are mandatory fields*.

**Table 3:** The data collected in the MDT form.

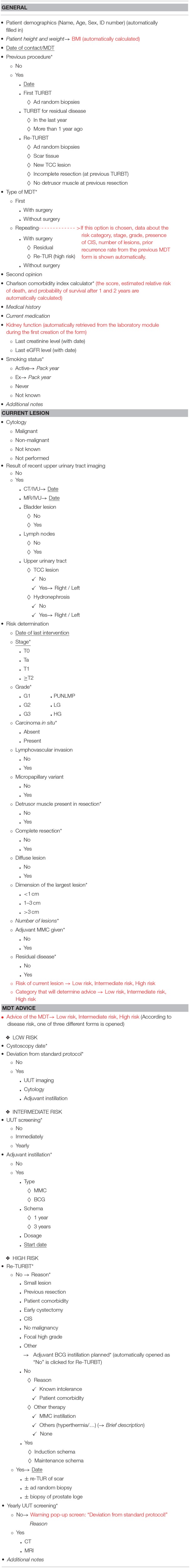

Underlined parameters are chosen from the drop-down menus. Parameters in italics need to be written manually. Automatically filled in and automatically calculated parameters are mentioned in parenthesis, and are colored in black and red, respectively. Answers for all other parameters are clicked from the options listed below the parameters. Items marked with an asterisk (*) are mandatory fields.

Biannual consensus meetings with members of the network (Vlaams Ziekenhuis Netwerk KU Leuven, VZNKul) were carried out to consent on reporting forms and discuss registry related topics. As such, a complete digital patient flow registration with scientific output parameters and with quality indicators based on the current knowledge has been developed. Patient flow-charts for the diagnosis of bladder cancer, and management of low-, intermediate- and high-risk NMIBCs are shown in Supplementary Figures 1–4.

Quality parameters for NMIBC management were identified through a comprehensive review of literature. For the review of quality parameters, a literature search including case control studies, cohort studies, randomized controlled trials (RCTs), systematic reviews and meta-analyses, was conducted on PubMed/Medline and Embase databases in March 2013. This comprehensive review has been renewed in March 2018, and was recently published (21). Currently selected quality indicators are listed in Table 4 (21, 22).

**Table 4:** Selected quality indicators.

Time between diagnosis of NMIBC and TURBT (percentage of patients that received surgery within 3 weeks) Percentage of patients that underwent complete resection Percentage of patients for whom the surgical report documents on visual completeness of the TURBT, depth of TURBT and examination under anesthesia findings Percentage of patients that undergoes adjuvant Mitomycin C instillation after complete TURBT Timing between surgery and instillation of adjuvant Mitomycin C (percentage of patients within 6 h and within 24 h) Percentage of pathology reports available within 1 week of TURBT Percentage of pathology reports noting detrusor muscle in pathologic specimen (for high-risk tumors) Percentage of newly diagnosed intermediate- and high-risk NMIBC patients that underwent upper tract imaging within 1 month before or after TURBT Percentage of patients with high-risk NMIBC and whose pathology report noted absence of detrusor muscle that underwent restaging TURBT within 6 weeks of initial resection Percentage of early recurrences Percentage of patients that started BCG, percentage of patients that completed 12 months of maintenance BCG Time between decision for early cystectomy and cystectomy

This registry was developed in accordance with the ethical standards laid down in the 1964 Declaration of Helsinki and its later amendments. The confidentiality of patient data was guaranteed. The registry protocol was first approved by the institutional review board (Clinical Trials Center [CTC] UZ Leuven) in August 2013. With amendments, the registry was finally approved by the Ethics Committee Research UZ/KU Leuven (approval date: 06/06/2014, approval number: S55725). According to the General Data Protection Regulation (GDPR), written informed consent is obtained from every included patient.

## Results

The first version of the registry was generated by using the standardized surgery report forms in August 2013 for all patients undergoing TURBT for NMIBC in four participating hospitals. Patient flow-charts for different risk categories were written and visualized on the intranet. With the addition of eCRF for bladder instillation in April 2016 and eCRF for MDT in September 2016, we started to use the second version of the registry system. The fifth to seventh hospital started enrolling patients in Q2 2017, Q1 2018, and Q1 2019, respectively. Several other hospitals are in the process of starting up the KWS system as a hospital-wide electronic patient file system and will start to enroll patients as soon as that process is completed. One of the four initial hospitals stopped recruiting patients after fusion with another non-participating center that is currently not using the same electronic patient file system.

The treating urologists make an update of the database while performing routine clinical practice. Written reports of the registered clinical actions are automatically generated and are incorporated in the medical file. When urologists complete these eCRFs, an automated ready-to-send letter to the general practitioner is generated. This automated letter motivates participating urologists to complete these eCRFs and ensures correct and complete data collection for all patients while providing an important time gain for the urologists. In all three forms, essential fields are mandatory to fill in, which ensures all relevant data to be collected properly. Automated pop-up windows warn the physician when a deviation from the standard-of-care management flow occurs.

Up till May 2019, 2756 TURBTs have been registered in 2,419 patients. The number of all TURBTs registered by each center according to registry version is listed in Supplementary Table 1. The numbers of all registered TURBTs and unique patients per each year are listed in Supplementary Table 2. The numbers of all registered bladder instillations and unique patients per year are listed in Supplementary Table 3.

The goal of the program is to continue the registry in order to have a large number of included patients with long follow-up. The power of the registry increases with time and with addition of other network hospitals using KWS database. Currently, we expect to collect clinical, pathological and outcome data for around 600 patients per year using eCRFs for TURBTs, bladder instillations and MDTs.

## Discussion

Collection of real-life data from cancer patients is a critical step of patient management and clinical science. Reliability due to accurately and timely collection of data, easiness to use the system and to evaluate the harvested data, and security of the stored data define the robustness of such a patient database. In the past, registration of patient data used to be a manual task. The number of qualitative registries is increasing with the implementation of electronic patient file systems, which allow easier and faster capture of data. Well-designed registries are a good way to collect and to analyze cancer survivorship in a real-life setting, and they have an added value next to randomized controlled studies (23–27). The population-based registries may either cover a region (California Cancer Registry), a country (SEER [Surveillance, Epidemiology, and End Results], NSQIP [The American College of Surgeons National Surgical Quality Improvement Program], SNRUBC [Swedish National Register of Urinary Bladder Cancer]) or a group of countries (EUROCARE-5).

EORTC and CUETO risk calculators tend to overestimate the risk of disease recurrence and progression in high-risk patients and have poor discrimination for prognostic outcomes in external validation. Recurrence and progression rates from current patients differ from those calculated from historical patient cohorts (15, 16), and therefore, need to be re-determined on patient cohorts that are categorized and treated according to the current state of the art. Based on the new data generated in this registry, we will try to address this by developing a new risk calculator, which will be readily available for Flemish hospitals to use.

NMIBC patients are often not treated according to the guidelines, because the management pathway given in these guidelines is complex, and establishment of a good patient flow is logistically difficult. We hypothesize that by standardizing the patient flow, especially with surgery report and MDT report, and monitoring it, these deviations from the guidelines-based follow-up will be structured and we can learn where and why these guidelines are not followed. Moreover, by rolling out the registry in different hospitals, we will find practice variation and be able to analyze (and eventually remediate) it.

The registry can be of direct value for the treatment of NMIBC patients in Flanders and on the long run even worldwide. We expect the eCRFs to have effect on three different but interrelated levels of the management of NMIBC:

1. Daily clinical practice: These eCRFs will ensure the urologists to make complete and standardized reporting of their patients, while decreasing their workload with easy-to-use style and automatically generated ready-to-send letters. Moreover, it will help to diminish deviations from standardized care paths. At the same time, each individual patient will benefit from this complete, standardized reporting and better risk stratification by having guidelines-based, state-of-the-art treatment. The registry will also provide high quality long-term follow-up data of the patients.
2. Centers: This registry will allow the participating centers to check their quality control of patient flow in NMIBC diagnosis and treatment, to monitor their adherence to the EAU guidelines, to detect internal practice variation, and to benchmark themselves with the other urology departments in regards of several outcome parameters and quality control parameters.
3. Knowledge of the disease: With the queries generated within the continuously growing real-life dataset, it will be able to reflect the current practice, to monitor guideline deviations and analyze them, to re-determine recurrence and progression rates, to serve as validation data for other calculators, and even to develop a novel risk calculator.

As long as TURBTs, MDTs and instillations are performed in the participating centers, the dataset will be continuously updated and enlarged. The treating urologist updates the database by merely writing the TURBT report, instillation report and MDT report. There is no extra action required. The number of included patients and follow-up will rapidly and highly exceed the current datasets used in the field. The EORTC and CUETO datasets have a median follow-up of 3.9 years and 69 months, respectively. From the initial start of the surgery registration as a pilot study (August 2013) up till May 2019, 2,756 TURBTs on 2,419 patients have already been included in the dataset. The long-term follow-up will allow more robust recurrence, progression and even survival data. Moreover, this registry can immediately be expanded to other centers that are using KWS in Flanders and to other (inter)national centers that are willing to use the same reporting standards. As such, the number of included patients per year is expected to increase in the years to come since more and more hospitals are joining healthcare networks.

The eCRFs include nine of the ten mandatory items (excluding tumor characteristics such as sessile, nodular, papillary, flat) and two of the three optional items (excluding separate deep biopsy sent from resection bed) of the checklist developed by Anderson et al. (11). The important features that make the registry unique are: (i) collection of all relevant clinical, pathological and follow-up data of the NMIBC patients, (ii) being completely implemented in the electronic patient file system, (iii) user friendly style with drop-down menus and clicking boxes used for the vast majority of the parameters and very few parameters to be entered by writing, iv) not missing data with all essential fields being mandatory, (v) warning the urologist with pop-up windows when a deviation from the standard of care occurs, and (vi) preparing a ready-to-send letter to the general practitioner that decreases the workload of the urologist. We think that this registry will ensure a better management of NMIBC patients while allowing us to collect robust and reliable data that can be used in various trials. Moreover, we are now building a pathology report to be filled in by pathologists for more detailed pathological reporting.

On the other hand, this registry is not devoid of limitations. First, it is readily available in KWS system, which may limit its use. However, it can be implemented into other electronic patient file systems with appropriate IT support. Second, automated queries can be performed for most of the quality parameters (21, 22), while a few ones have to be queried manually. This deficit can be compensated by an improvement in the software. And last, it currently includes only NMIBC patients, however, we are in the process of developing eCRFs for MIBC patients.

## Conclusion

The easy-to-use eCRFs, which generate automated letters to general practitioners, harvest important data that will be used to define real-life data of NMIBC patients, to challenge the existing guidelines, to create a novel risk stratification tool, and to monitor the quality parameters for NMIBC patient flow. We hope that this registry can be disseminated to more urology departments in the near future, and also sets a precedent to further registries in different departments/diseases.

## Data Availability Statement

The datasets generated for this study are available on request to the corresponding author.

## Ethics Statement

This registry was developed in accordance with the ethical standards laid down in the 1964 Declaration of Helsinki and its later amendments. The confidentiality of patient data was guaranteed. The registry protocol was first approved by the institutional review board (Clinical Trials Center [CTC] UZ Leuven) in August 2013. With amendments, the registry was finally approved by the Ethics Committee Research UZ/KU Leuven (approval date: 06/06/2014, approval number: S55725). According to the General Data Protection Regulation (GDPR), written informed consent is obtained from every included patient.

## Author Contributions

FV, MA, TM, JC, SV, and KV contributed to the conception and design of the study. FV, JC, SV, KV, FB, PM, RV, and BV organized the database. MA and TM wrote the first draft of the manuscript. FV and SJ edited the manuscript. All authors contributed to manuscript revision, read, and approved the submitted version.

### Conflict of Interest

The authors declare that the research was conducted in the absence of any commercial or financial relationships that could be construed as a potential conflict of interest.

## Supplementary Material

Supplementary Figure 1 Patient flow-chart for the diagnosis of bladder cancer and treatment with TURBT. NMIBC, Non-muscle-invasive bladder cancer; eGFR, Estimated glomerular filtration rate; CT IVU, Computed tomography – intravenous urography; UUT TTC, Upper urinary tract transitional cell carcinoma; TURBT, Transurethral resection of bladder tumor; MRI, Magnetic resonance imaging; MMC, Mitomycin C; MDT, Multidisciplinary team; CIS, Carcinoma in situ; LG, Low grade; MIBC, Muscle-invasive bladder cancer.

Supplementary Figure 2 Patient flow-chart for the management low-risk of bladder cancer. MDT, Multidisciplinary team; TURBT, Transurethral resection of bladder tumor.

Supplementary Figure 3 Patient flow-chart for the management intermediate-risk of bladder cancer. MDT, Multidisciplinary team; TURBT, Transurethral resection of bladder tumor; UUT, Upper urinary tract; CT IVU, Computed tomography – intravenous urography; MMC, Mitomycin C; BCG, Bacillus Calmette-Guérin.

Supplementary Figure 4 Patient flow-chart for the management high-risk of bladder cancer. MIBC, Muscle-invasive bladder cancer; CIS, Carcinoma in situ; BCG, Bacillus Calmette-Guérin; TURBT, Transurethral resection of bladder tumor; ADR, Ad random biopsy; MDT, Multidisciplinary team; UUT, Upper urinary tract.

Supplementary Table 1 The distribution of the TURBTs registered by each center according to registry version (till the beginning of May 2019).

Supplementary Table 2 The number of all TURBTs and unique patients per year.

Supplementary Table 3 The number of all bladder instillations and unique patients per year.
