# Supplementary figures and images for: Development of a Prospective Data Registry System for Non-muscle-Invasive Bladder Cancer Patients Incorporated in the Electronic Patient File System
Source: Front Oncol. 2019 Dec 11;9:1402. doi: 10.3389/fonc.2019.01402 (PMC6917611; doi:10.3389/fonc.2019.01402)

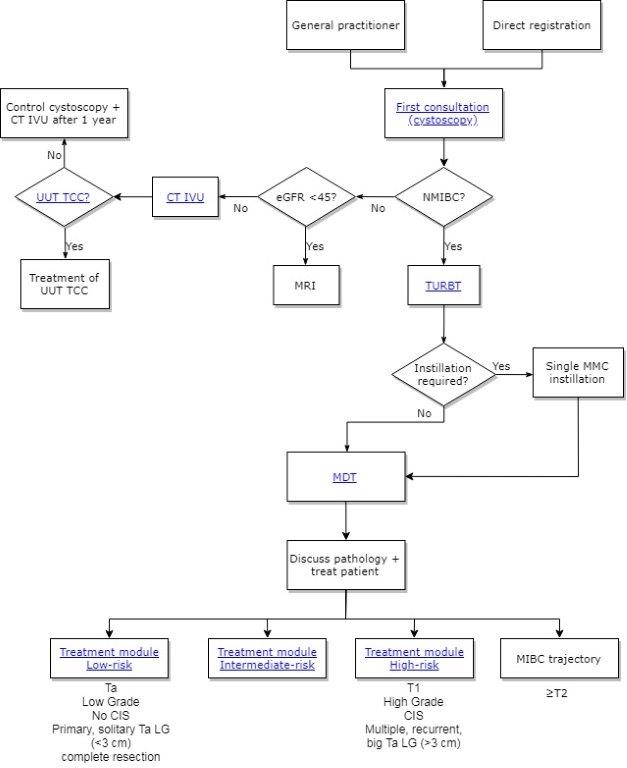

Supplement: Supplementary Figure 1 — Patient flow-chart for the diagnosis of bladder cancer and treatment with TURBT. NMIBC, Non-muscle-invasive bladder cancer; eGFR, Estimated glomerular filtration rate; CT IVU, Computed tomography – intravenous urography; UUT TTC, Upper urinary tract transitional cell carcinoma; TURBT, Transurethral resection of bladder tumor; MRI, Magnetic resonance imaging; MMC, Mitomycin C; MDT, Multidisciplinary team; CIS, Carcinoma in situ; LG, Low grade; MIBC, Muscle-invasive bladder cancer. [file Image_1.JPEG]

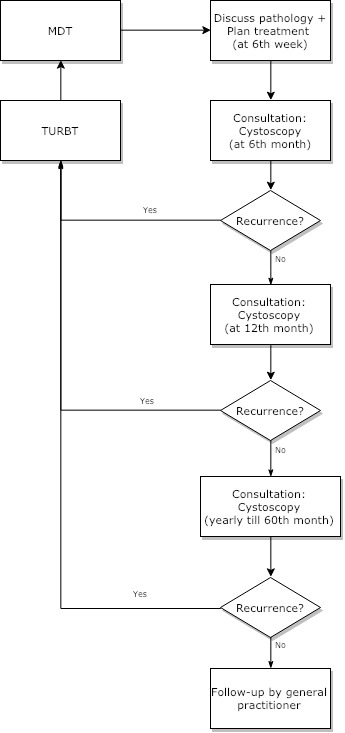

Supplement: Supplementary Figure 2 — Patient flow-chart for the management low-risk of bladder cancer. MDT, Multidisciplinary team; TURBT, Transurethral resection of bladder tumor. [file Image_2.JPEG]

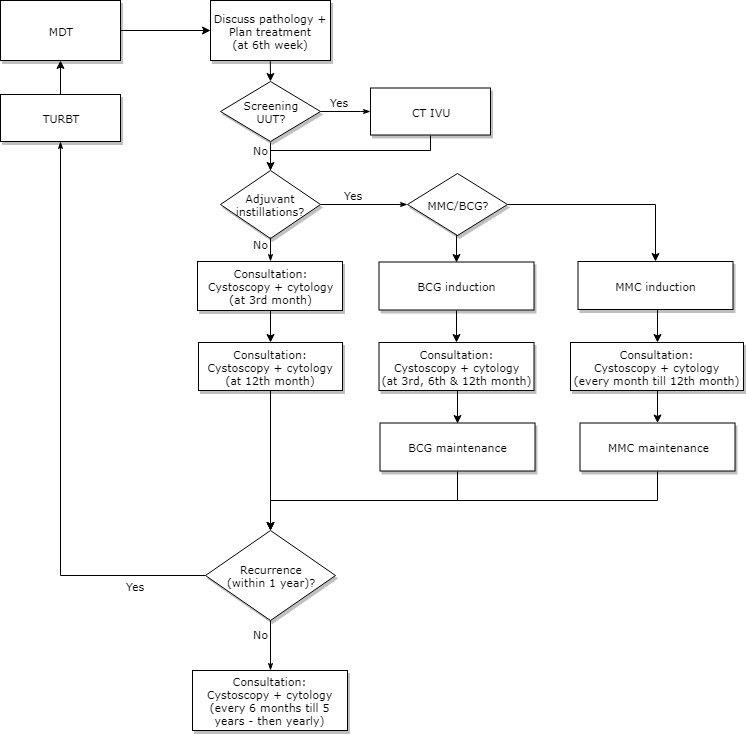

Supplement: Supplementary Figure 3 — Patient flow-chart for the management intermediate-risk of bladder cancer. MDT, Multidisciplinary team; TURBT, Transurethral resection of bladder tumor; UUT, Upper urinary tract; CT IVU, Computed tomography – intravenous urography; MMC, Mitomycin C; BCG, Bacillus Calmette-Guérin. [file Image_3.JPEG]

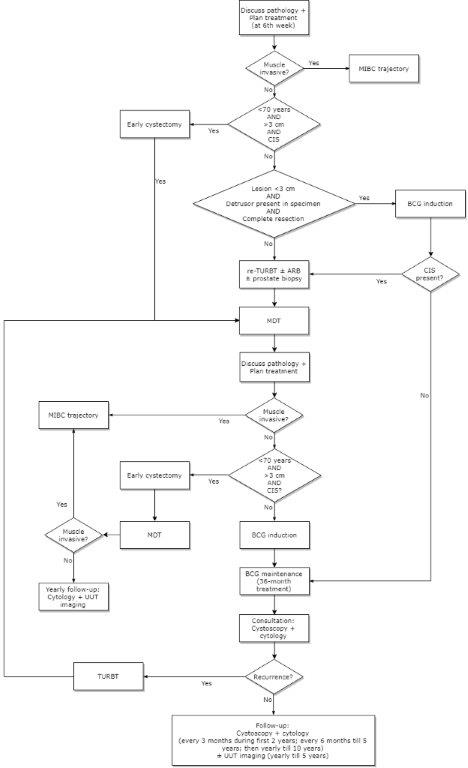

Supplement: Supplementary Figure 4 — Patient flow-chart for the management high-risk of bladder cancer. MIBC, Muscle-invasive bladder cancer; CIS, Carcinoma in situ; BCG, Bacillus Calmette-Guérin; TURBT, Transurethral resection of bladder tumor; ADR, Ad random biopsy; MDT, Multidisciplinary team; UUT, Upper urinary tract. [file Image_4.JPEG]
