# Supplementary material for: Development of a Prospective Data Registry System for Non-muscle-Invasive Bladder Cancer Patients Incorporated in the Electronic Patient File System
Source: Front Oncol. 2019 Dec 11;9:1402. doi: 10.3389/fonc.2019.01402 (PMC6917611; doi:10.3389/fonc.2019.01402)
Supplement: Supplementary Table 1 — The distribution of the TURBTs registered by each center according to registry version (till the beginning of May 2019). [file Table_1.DOCX]

**Supplementary Table 1:** The distribution of the TURBTs registered by each center according to registry version (till the beginning of May 2019).

| **Center name** | **Center code** | **Version 1** | **Version 2** | **Total** |
| --- | --- | --- | --- | --- |
| Leuven | URO | 545 | 831 | 1376 |
| Kortrijk | KURO | 262 | 390 | 652 |
| Dendermonde | EURO | 183 | 329 | 512 |
| Diest | DURO | 30 | 3 | 33 |
| Oostende | NURO | - | 76 | 76 |
| Brugge | BURO | - | 82 | 82 |
| Turnhout | WURO | - | 25 | 25 |
| **Total** | | **1020** | **1736** | **2756** |
