# Supplementary material for: Development of a Prospective Data Registry System for Non-muscle-Invasive Bladder Cancer Patients Incorporated in the Electronic Patient File System
Source: Front Oncol. 2019 Dec 11;9:1402. doi: 10.3389/fonc.2019.01402 (PMC6917611; doi:10.3389/fonc.2019.01402)
Supplement: Supplementary Table 2 — The number of all TURBTs and unique patients per year. [file Table_2.DOCX]

**Supplementary Table 2:** The number of all TURBTs and unique patients per year.

| **Year** | **Operation Number** | **Unique Patient Number** |
| --- | --- | --- |
| 2013 | 121 | 116 |
| 2014 | 296 | 270 |
| 2015 | 383 | 346 |
| 2016 | 479 | 424 |
| 2017 | 553 | 465 |
| 2018 | 649 | 539 |
| 2019* | 275 | 259 |
| **Total** | **2756** | **2419** |

* Till the beginning of May 2019
