# Supplementary material for: Development of a Prospective Data Registry System for Non-muscle-Invasive Bladder Cancer Patients Incorporated in the Electronic Patient File System
Source: Front Oncol. 2019 Dec 11;9:1402. doi: 10.3389/fonc.2019.01402 (PMC6917611; doi:10.3389/fonc.2019.01402)
Supplement: Supplementary Table 3 — The number of all bladder instillations and unique patients per year. [file Table_3.DOCX]

**Supplementary Table 3:** The number of all bladder instillations and unique patients per year.

| **Year** | **Instillation Number** | **Unique Patient Number** |
| --- | --- | --- |
| 2016 | 516 | 108 |
| 2017 | 773 | 127 |
| 2018 | 628 | 94 |
| 2019* | 184 | 52 |
| **Total** | **2101** | **381** |

* Till the beginning of May 2019
